# Impact of Therapy in Patients with Hematologic Malignancies on Seroconversion Rates After SARS-CoV-2 Vaccination

**DOI:** 10.1093/oncolo/oyac032

**Published:** 2022-03-11

**Authors:** Deniz C Guven, Taha K Sahin, Serkan Akın, Fatih M Uckun

**Affiliations:** 1 Department of Medical Oncology, Hacettepe University Cancer Institute, Ankara, Turkey; 2 Department of Internal Medicine, Hacettepe University Faculty of Medicine, Ankara, Turkey; 3 Bone Marrow Transplantation Unit, Hacettepe University Cancer Institute, Ankara, Turkey; 4 Immuno-Oncology Program and COVID-19 Task Force, Ares Pharmaceuticals, St. Paul, MN 55110, USA

**Keywords:** antibody, COVID-19, hematological malignancies, SARS-CoV-2, vaccine

## Abstract

**Introduction:**

The leading professional organizations in the field of hematology have recommended severe acute respiratory syndrome coronavirus 2 (SARS-Cov-2) vaccination for all patients with hematologic malignancies notwithstanding efficacy concerns. Here we report a systematic literature review regarding the antibody response to SARS-CoV-2 vaccination in patients with hematologic malignancies and its key determinants.

**Methods:**

We conducted a systematic search of original articles evaluating the seroconversion rates with SARS-CoV-2 vaccines in hematological malignancies from the PubMed database published between April 1, 2021 and December 4, 2021. Calculated risk differences (RD) and 95% confidence intervals (CI) to compare seroconversion rates between patients with hematologic malignancies versus healthy control subjects used the Review Manager software, version 5.3.

**Results:**

In our meta-analysis, we included 26 studies with control arms. After the first dose of vaccination, patients with hematologic malignancies had significantly lower seroconversion rates than controls (33.3% vs 74.9%; RD: −0.48%, 95% CI: −0.60%, −0.36%, *P* < .001). The seroconversion rates increased after the second dose, although a significant difference remained between these 2 groups (65.3% vs 97.8%; RD: −0.35%, 95% CI: −0.42%, −0.28%, *P* < .001). This difference in seroconversion rates was particularly pronounced for Chronic Lymphocytic Leukemia (CLL) patients (RD: −0.46%, 95% CI: −0.56, −0.37, *P* < .001), and for patients with B-lineage leukemia/lymphoma treated with anti-CD20 antibodies (RD: −0.70%, 95% CI: −0.88%, −0.51%, *P* < .001) or Bruton Tyrosine Kinase Inhibitors (BTKi; RD: −0.63%, 95% CI: −0.85%, −0.41%, *P* < .001). The RD was lower for patients under remission (RD: −0.10%, 95% CI: −0.18%, −0.02%, *P* = .01).

**Conclusion:**

The seroconversion rates following SARS-CoV-2 vaccination in patients with hematologic malignancies, especially in CLL patients and patients treated with anti-CD20 antibodies or BTKi, were significantly lower than the seroconversion rates in healthy control subjects. Effective strategies capable of improving vaccine efficacy in these vulnerable patient populations are urgently needed.

## Introduction

The vaccination against severe acute respiratory syndrome coronavirus 2 (SARS-Cov-2) emerged as the primary strategy in the fight against the COVID-19 pandemic, and available vaccines have decreased COVID-19 mortality and morbidity worldwide.^1-4^ Patients with hematological malignancies were prioritized for the vaccination against SARS-CoV-2,^5^ considering the high rate of mortality and morbidity of COVID-19 in these vulnerable patient populations.^6^ Early reports have suggested significantly decreased antibody responses to SARS-CoV-2 vaccination in patients with hematologic malignancies, although the sample sizes, treatment factors, and included patient cohorts were heterogeneous.^7-9^ Therefore, we systemically reviewed the available data on the antibody response to SARS-CoV-2 vaccination in patients with hematologic malignancies in the context of disease status and immunosuppressive therapy.

## Methods

### Literature Search

We conducted a systematic review from the PubMed database per the PRISMA guidelines^10^ with MeSH terms: “vaccine” OR “vaccination” AND “cancer” OR “malignancy” OR “neoplasms” OR “myeloid” OR “myeloma” OR “leukemia” OR “leukaemia” OR “lymphoma” OR “hematological” OR “myeloproliferative”. We included original articles evaluating the seroconversion rates with SARS-CoV-2 vaccines in hematological malignancies published between April 1, 2021 and December 4, 2021.

### Study Selection and Meta-Analyses

Our systematic search retrieved a total of 5261 records and we included 26 studies with control arms in the analyses (12 studies for first dose and 22 studies for second dose) (Supplementary Fig. S1).

We performed meta-analyses via generic inverse-variance method with a random-effects model and reported heterogeneity with the I-square statistics. The principal summary measure was the risk difference (RD) with 95% 2-sided confidence intervals (CI). All analyses were done using the Review Manager software, version 5.3 (The Nordic Cochrane Center, The Cochrane Collaboration, Copenhagen, Denmark). The *P* values of <.05 were considered statistically significant.

## Results

### Seroconversion Rates After First Vaccination

**Figure 1.**
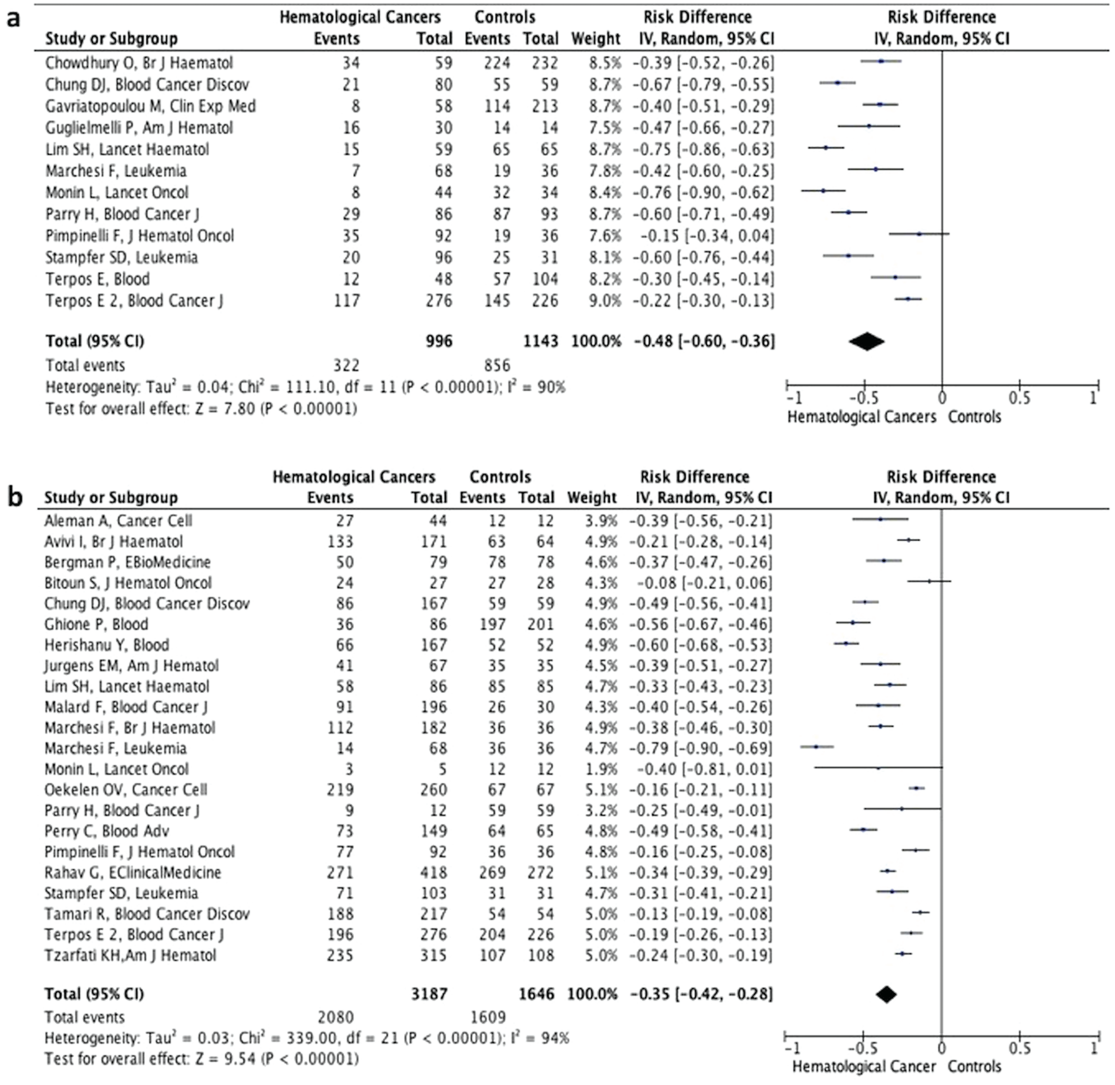
Forest plot illustrating the risk differences of seroconversion rates between patients with hematologic malignancies and healthy controls with first dose of vaccination **(a)** and second dose of vaccination **(b)**.

Low seroconversion rates after the first vaccine dose as a consistent finding across all studies^7,9,11-21^ (Supplementary Table S1), represented a sharp contrast to over 80% seroconversion rates after the first vaccine dose in healthy control groups of most studies (8/12).^9,11,13,15,16,18-20^ In the pooled data from 12 studies, patients with hematologic malignancies had significantly lower likelihood of seroconversion after the first dose of vaccination (322/996, 33.3%) than healthy controls (856/1143, 74.9%; RD: −0.48%, 95% CI: −.60%, −0.36%, *P* < .001; Supplementary Table S1 and Fig. 1a). Significant variability existed among the studies (*I*^2^ = 90%; Fig. 1a). Sensitivity analyses by subtracting individual studies from the equation showed a consistent negative effect.

### Seroconversion Rates After Second Vaccination

**Figure 2.**
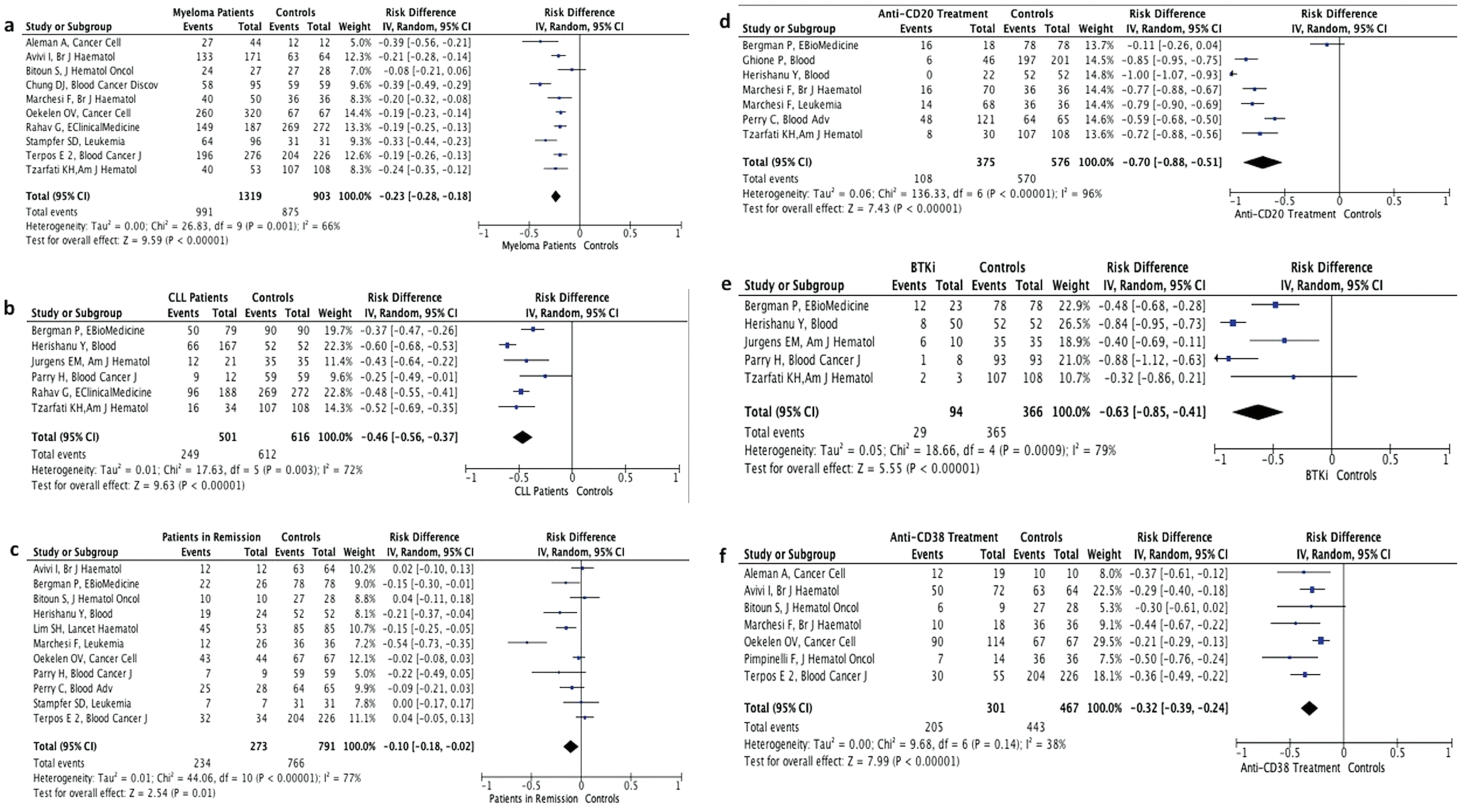
Forest plot illustrating the risk differences of seroconversion rates between multiple myeloma patients **(a),** CLL patients **(b)**, patients in remission **(c)**,hematologic malignancy patients treated with anti-CD 20 monoclonal antibodies **(d)**, BTK inhibitors **(e)**, anti-CD38 monoclonal antibodies **(f)**, and healthy controls after second dose of vaccination.

In the pooled analysis of 22 studies encompassing 3187 patients,^7,9,11,16-18,20-35^ the possibility of an antibody response to 2-dose vaccination was 35% lower in patients with hematologic malignancies (97.8% in the control arms vs 65.3% in hematological malignancies) (RD: −0.35%, 95% CI: −0.42%, −0.28%, *P* < .001; Fig. 1b). Additionally, the antibody titers were consistently lower in patients with hematologic malignancies than healthy controls in most studies (Supplementary Table S2). The difference in seroconversion rates was most pronounced in Chronic Lymphocytic Leukemia (CLL) patients (RD: −0.46%, 95% CI: −0.56, −0.37, *P* < .001) (Fig. 2b), while the difference in seroconversion rates was lower in myeloma patients compared with controls (RD: −0.23%, 95% CI: −0.28, −0.18, *P* < .001) (Fig. 2a).

### The Effects of Treatments on Seroconversion Rates with 2-Dose Vaccination

Eleven studies reported specific outcomes for patients in remission^11,16-18,21-25,30,31^ (Supplementary Table S2). In the pooled analysis of these studies, the patients in remission had lower seroconversion rates than healthy controls, although with a smaller RD (RD: −0.10%, 95% CI: −0.18%, −0.02%, *P* = .01; Fig. 2c). In contrast, the seroconversion rates after 2-dose vaccination were strikingly lower in B-lineage leukemia/lymphoma patients treated with anti-CD20 antibodies (RD: −0.70%, 95% CI: −0.88%, −0.51%, *P* < .001; Fig. 2d) or Bruton Tyrosine Kinase Inhibitors (BTKi; RD: −0.63%, 95% CI: −0.85%, −0.41%, *P* < .001) compared with controls (Fig. 2e). Patients treated with an anti-CD38 antibody also had lower seroconversion rates with 2 dose vaccination (RD: −0.32%, 95% CI: −0.39%, −0.24%, *P* < .001; Fig. 2f). Significant heterogeneity was present in all analyses (Figs. 1a and b and 2a-f).

## Discussion

In this meta-analysis, we consistently observed significantly lower seroconversion rates in patients with hematologic malignancies compared with healthy controls after 2-dose vaccination. Treatment with an anti-CD20 antibody or a BTKi appeared to accentuate this difference. However, several questions remain unanswered.

First, a consistently effective strategy for patients who remain seronegative after 2-dose vaccination is yet to be deciphered. In August 2021, FDA recommended a third-dose booster to immunosuppressive patients and later expanded this recommendation to individuals over 18 years of age with a high-risk of severe COVID-19 disease due to population level data. However, the efficacy of the third-dose booster is relatively unknown in hematologic malignancies. In a recent study, 18 of 18 seronegative patients with lymphoid malignancies remained seronegative after a third vaccine dose.^36^ Similarly, Marchesi et al observed only 4 seroconversions with a third-dose booster in 50 seronegative B-cell NHL patients.^21^ Likewise, whether the T-cell immunity correlates with antibody responses to vaccination is unknown. The observation of T-cell responses in the absence of seroconversion in approximately 25% of seronegative patients and a higher rate of T-cell responses than antibody responses in patients treated with anti-CD20 agents warrants measuring and reporting T-cell responses in addition to seroconversion in patients with hematologic malignancies.^37^

Another vital question is the clinical efficacy of the COVID-19 vaccines. The clinical efficacy was the main endpoint of vaccine clinical trials, although real-life studies reported mostly seroconversion rates. Mittelman et al reported significantly higher risk of COVID-19 infection (RR 1.60, 95% CI: 1.12-2.37), severe COVID-19 infection (RR 2.27, 95% CI 1.18-5.19), and COVID-19-related deaths (RR 1.66, 95% CI 0.72-4.47) in vaccinated patients with hematologic malignancies compared with general population.^38^ Similarly, Heudel et al reported significantly higher mortality rates in patients with hematologic malignancies in a cohort of 1503 patients with cancer.^39^ These data further motivates applying additional boosters and priorization of passive immunization strategies for patients with hematologic cancers.

Finally, the present study mostly reported outcomes with mRNA vaccines and the data on efficacy of vaccines other than mRNA vaccines are scarce. However, several regions of the World are using different vaccines. There is a need for additional studies evaluating the efficacy of other available vaccines in patients with hematologic malignancies.

## Supplementary Material

oyac032_suppl_Supplementary_Figure_1

oyac032_suppl_Supplementary_Table_1

oyac032_suppl_Supplementary_Table_2

## Data Availability

The data underlying this article will be shared on reasonable request to the corresponding author.
