# Supplementary figures and images for: Impact of Therapy in Patients with Hematologic Malignancies on Seroconversion Rates After SARS-CoV-2 Vaccination
Source: Oncologist. 2022 Mar 11;27(4):e357–61. doi: 10.1093/oncolo/oyac032 (PMC8982368; doi:10.1093/oncolo/oyac032)

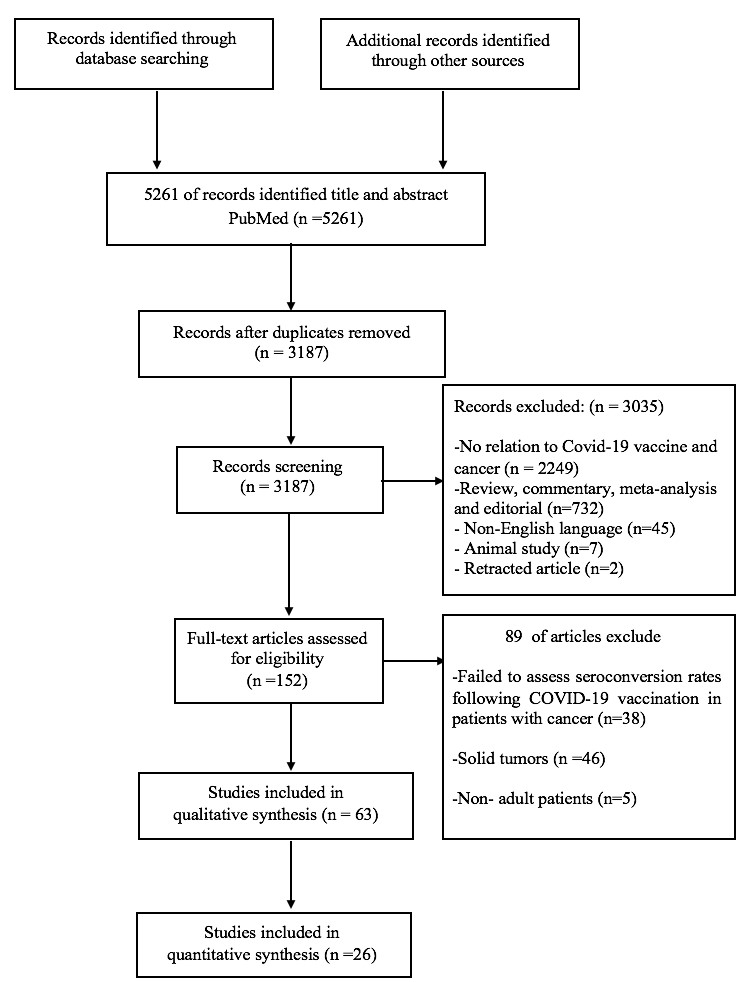

Supplement: oyac032_suppl_Supplementary_Figure_1 [file oyac032_suppl_supplementary_figure_1.jpeg]
