# Supplementary material for: Impact of Therapy in Patients with Hematologic Malignancies on Seroconversion Rates After SARS-CoV-2 Vaccination
Source: Oncologist. 2022 Mar 11;27(4):e357–61. doi: 10.1093/oncolo/oyac032 (PMC8982368; doi:10.1093/oncolo/oyac032)
Supplement: oyac032_suppl_Supplementary_Table_1 [file oyac032_suppl_supplementary_table_1.docx]

**Supplemental Table-1. Summary of Studies Included in Meta-analysis Evaluating Antibody Responses to First Dose SARS-CoV-2 Vaccination**

| **Study** | **Patient Cohort** | **Vaccine type** | **Number of Participants** | **Baseline Antibody Measurement** | **Antibody Assay and Threshold** | **Antibody Response Of Patients After 1^st^ Dose** | **Antibody Response of Control Group After 1^st^ Dose** | **Antibody Response of Patients in Remission After 1^st^ Dose** | **Antibody Response of**  **Patients Under Active Treatment After 1^st^ Dose** | **The Frequency of Systemic Adverse Effects After 2^nd^ Dose / The frequency of Local Adverse Effects After 1^st^ Dose** | **Additional Findings** | **Reference** |
| --- | --- | --- | --- | --- | --- | --- | --- | --- | --- | --- | --- | --- |
| Monin L, Lancet Oncol | Mature B-cell neoplasms, mature T-cell neoplasms, acute leukaemia, myeloid neoplasms, amyloidosis and Erdheim-Chester disease | BNT162b2(Pfizer-BioNTech) | 56 Patients/54 Controls | Negative SARS-CoV-2 S/Negative rRT-PCR | Anti-SARS-CoV-2 S-specific IgG : ≥70 EC_50_ dilution units | 18% | 94% | N/A | N/A | 10% / 21 % | T-cell responses in 82%, 71% and 50% of the controls, solid tumor cohort and hematologic tumor cohort with first vaccine dose | [9] |
| Pimpinell F, J Hematol Oncol | MM/MPN | BNT162b2(Pfizer-BioNTech) | 92 Patients ( 42 MM 50 MPN/36 Controls | SARS-CoV-2 S1/S2 IgG test | LIAISON® SARS-CoV-2 S1/S2 IgG by DiaSorin®, Saluggia, Italy : ≥15 AU/ml | 21.4% in myeloma/52% in MPN | 52.8% | N/A | N/A | 7% /30% | No statistical difference between MPM patients and control cohort( p=0.837) | [7] |
| Lim SH,J  Lancet Haematol | Lymphoma | BNT162b2(Pfizer-BioNTech)  and AZD1222(Oxford–AstraZeneca) | 119 Patients/ 150 Controls | Negative anti-SARS-CoV-2 nucleocapsid  protein IgG | Qualified electrochemiluminescent Anti-SARS-CoV-2 S assay (Meso Scale Discovery, Rockville, MD, USA): >0.55 BAU/ml | 32.2% | 100% | N/A | N/A | N/A | Lower seroconversion rates in patients who received systemic anti-lymphoma therapy after the first BNT162b2 vaccine dose (p= 0.0002) | [11] |
| Terpos E, Blood | MM | BNT162b2(Pfizer-BioNTech) | 48 Patients/104 Controls | Neutralizing  Antibodies Against SARS-CoV-2 | Neutralizing antibody assay Genscript:  ≥30% positive; ≥ 50% clinically relevant | 25% | 54.8% | 100% | 22.8% | N/A | All patients with clinically relevant viral inhibition (%4/4) after first dose was in remission without treatment | [12] |
| Chowdhury O, Br J Haematol | CMN | BNT162b2(Pfizer-BioNTech)  or AZD1222(Oxford–AstraZeneca) | 59 Patients/ 232 Controls | Negative anti-SARS-CoV-2 nucleocapsid  protein IgG | Abbott SARS-CoV-2 IgG II Quant Assay: ≥50 AU/ml | 58% | 97% | 65% | %0 – Ruxolitinib  36% - hydroxycarbamide  88% - İnterferon | N/A | The highest seroconversion rates in patients with CML (75%) | [13] |
| Gavriatopoulou M, Clin Exp Med. | WM, CLL and NHL | BNT162b2(Pfizer-BioNTech)  and AZD1222(Oxford–AstraZeneca) | 58 Patients/ 213 Controls | N/A  (No History of COVID-19) | Neutralizing antibody assay cPass: ≥ 30% positive, ≥ 50% clinically relevant inhibition | 14% | %54 | N/A | 0% | N/A | Lower response rates (< 30%) in patients under active treatment (17/37) | [14] |
| Diefenbach C, medRxiv | CLL, HL and NHL | BNT162b2(Pfizer-BioNTech)  and mRNA-1273 (Moderna) | 53 Patients/ 5 Controls | N/A | Multiplex bead-binding IgG spike and receptor binding domain assay for SARS-CoV2 : in house assay mean plus 3 x SD of median fluorescence intensities | N/A | 100% | N/A | N/A | N/A | Lower seroconversion rates in patients treated with anti-CD20 (p<0.001) and BTK inhibitors (p=0.003)/No effect of additional boost on antibody titers in most patients (94%) | [15] |
| Parry, H.  *Blood Cancer J* | CLL | BNT162b2(Pfizer-BioNTech)  and AZD1222(Oxford–AstraZeneca) | 286 Patients/ 93 Controls | Negative anti-SARS-CoV-2 nucleocapsid  protein IgG | Serum Samples--Elecsys^®^ Anti-SARS-CoV-2 S ECLIA : ≥0.8 IU/ml  Dried blood samples- SARS-CoV-2 spike- specific antibody responses | 34% - Serum samples  24%- DBS | 94 % - Serum samples  71%-DBS | N/A | 14.8% (BTK inhibitors)  0% ( Venetoclax) | N/A | Lower seroconversion rates in patients treated with BTK inhibitors compared to those on watch and wait after the first vaccine dose (p=0.0056) | [16] |
| Terpos E, *Blood Cancer J*. | MM | BNT162b2(Pfizer-BioNTech)  and AZD1222(Oxford–AstraZeneca) | 276 Patients (MM  213 , SMM 38, and MGUS 25)  / 226 Controls | Neutralizing  Antibodies Against SARS-CoV-2 | Neutralizing antibody assay Genscript:  ≥30% positive; ≥ 50% clinically relevant | 42.4% | 64.2% | N/A | N/A | 13% / 33% | Lower Nab responses in MM patients  compared with MGUS after first vaccine dose (p = 0.009) | [17] |
| Stampfer SD, *Leukemia*. | MM | BNT162b2(Pfizer-BioNTech)  and mRNA-1273 (Moderna) | 103 Patients( 96 MM and 7 SMM) / 31 Controls | Anti-SARS-CoV-2  spike IgG | Anti-SARS-CoV-2  spike IgG : 50-250 IU/ml partial response,  >250 IU/ml clinically relevant response | 21% | 81% | N/A | 21% | N/A | No statistical difference in spike antibody levels between patients who received first dose of mRNA-1273 and BNT162b2 vaccine | [18] |
| Guglielmelli, P.,*Am J Hematol.* | MPN | BNT162b2(Pfizer-BioNTech)  and mRNA-1273 (Moderna) | 30 patients ( 10 PV, 7 ET, 13 MF / 14 controls | Negative anti-SARS-CoV-2 nucleocapsid  antibodies | Anti-S IgG, Anti RBD IgG and neutralizing antibodies : N/A | 53.3% | 100% | 91.6 | 33.3 | N/A | Lower seroconversion rates in patients treated with ruxolitinib after the first vaccine dose (p=0.001) | [19] |
| Chung DJ, *Blood Cancer Discov* | Leukemia, Lymphoma and MM | BNT162b2(Pfizer-BioNTech)  and mRNA-1273 (Moderna) | 551 Patients(157 Leukemia, 173 Lymphoma and 221 MM) / 69 Controls | Anti–SARS-CoV-2 spike IgG antibody | Anti–SARS-CoV-2 spike IgG immunoassay : ≥50.0 AU/mL | 26.3% | 93.2% | N/A | N/A | N/A | Lower seroconversion rates in patients treated with  BTK inhibitors, venetoclax, anti-CD20–directed therapies, and anti-CD38/BCMA–directed therapies. | [20] |
| Marchesi F, Leukemia | NHL | BNT162b2(Pfizer-BioNTech) | 68 Patients/ 36 Controls | Anti SARS-CoV-2 S1/S2 IgG | Anti SARS-CoV-2 S1/S2 IgG Liaison: ≥15 U/m | 10.2% | 52.8% | N/A | N/A | N/A | No statistical difference was observed between patients on follow-up with last dose of anti-CD20 administered beyond 3 months before vaccination and the control group | [21] |

**Abbreviations:** BCMA: B-cell maturation antigen; BTK: Bruton tyrosine kinase; CML: Chronic Myeloid Leukaemia; CLL: Chronic lymphocytic leukemia; COVID-19, Coronavirus disease 2019; DBS: Dried blood samples; ET:Essential thrombocythemia; HL: Hodgkin Lymphoma; MGCS: Monoclonal gammopathies of clinical significance; MGUS: Monoclonal Gammopathy of Undetermined Significance; MF: Myelofibrosis; MM: Multiple myeloma; MPN: Myeloproliferative neoplasms; N/A: Not applicable; NHL: Non-Hodgkin Lymphoma; PV: Polycythemia vera; RBD(receptor binding domain); SARS-CoV-2: Severe Acute Respiratory Syndrome Coronavirus-2; SMM: Smoldering Multiple Myeloma; WM: Waldenstrom macroglobulinemia
