# Supplementary material for: Impact of Therapy in Patients with Hematologic Malignancies on Seroconversion Rates After SARS-CoV-2 Vaccination
Source: Oncologist. 2022 Mar 11;27(4):e357–61. doi: 10.1093/oncolo/oyac032 (PMC8982368; doi:10.1093/oncolo/oyac032)
Supplement: oyac032_suppl_Supplementary_Table_2 [file oyac032_suppl_supplementary_table_2.docx]

**Supplemental Table-2. Summary of Studies Included in Meta-analysis Evaluating Antibody Responses to Second Dose SARS-CoV-2 Vaccination**

| **Study** | **Patient Cohort** | **Vaccine type** | **Number of Participants** | **Baseline Antibody Measurement** | **Antibody Assay and Threshold** | **Antibody Response Of Patients After 2^nd^ Dose** | **Antibody Response of Control Group After 2^nd^ Dose** | **Antibody Response of Patients in Remission After 2^nd^ Dose** | **Antibody Response of**  **Patients Under Active Treatment After 2^nd^ Dose** | **The Frequency of Systemic Adverse Effects After 2^nd^ Dose / The frequency of Local Adverse Effects After 2^nd^ Dose** | **Additional Findings** | **Reference** |
| --- | --- | --- | --- | --- | --- | --- | --- | --- | --- | --- | --- | --- |
| Herishanu Y, Blood | CLL | BNT162b2(Pfizer-BioNTech) | 167 Patients (52 Patient and  52 Control for Matched Cohort) | N/A  (No History of COVID-19) | Elecsys Anti–SARS-CoV-2 Spike IgG: ≥0.8 IU/ml | 39.5% | 100% | 79.1% | 16 %  0%- Anti CD 20 Treatment | 23.4% /33.5% | Lower seroconversion in patients under treatment (16%) vs. patients with clinical remission (79.2%) and treatment-naïve patients (55.2%)/ No seroconversion in patients exposed to anti-CD20 treatment in last 12 months (0/22) | [23] |
| Oekelen OV, Cancer Cell | MM | BNT162b2(Pfizer-BioNTech)  and mRNA-1273 (Moderna) | 320 Patients/67 Controls | N/A (Prior COVID-19 infection in 60 patients) | COVID-SeroKlir Kantaro  SARS-CoV-2 IgG test:  ≥5 AU/ml | 81.25% | 100% | 97.7% | 81.9%  78.9 %- Anti CD38 Treatment | N/A | Lower seroconversion rates in patients treated with anti-CD38 (HR: 4.258, p=0.005) or BCMA-targeted treatment (HR: 10.269, p<0.001)/Better seroconversion rates in patients with CR (HR: 0.389, p=0.037) | [24] |
| Monin L, Lancet Oncol | Mature B-cell neoplasms, mature T-cell neoplasms, acute leukaemia, myeloid neoplasms, amyloidosis and Erdheim-Chester disease | BNT162b2(Pfizer-BioNTech) | 56 Patients/54 Controls | Negative SARS-CoV-2 S/Negative rRT-PCR | Anti-SARS-CoV-2 S-specific IgG : ≥70 EC_50_ dilution units | 60% | 100% | N/A | N/A | 7% / 16% | T-cell responses in 100%, 88% and 75% of the controls, solid tumor cohort and hematologic tumor cohort with second vaccine dose | [9] |
| Pimpinell F, J Hematol Oncol | MM/MPN | BNT162b2(Pfizer-BioNTech) | 92 Patients/36 Controls | SARS-CoV-2 S1/S2 IgG test | LIAISON® SARS-CoV-2 S1/S2 IgG by DiaSorin®, Saluggia, Italy : ≥15 AU/ml | 78.6% in myeloma/88% in MPN | 100% | N/A | N/A | 10% / 26 % | Lower seroconversion rates in daratumumab-treated patients (50% vs. 92.9%, p=0.003)/ | [7] |
| Avivi I,  Br J Haematol | MM | BNT162b2(Pfizer-BioNTech) | 171 Patients/64 Controls | Negative anti-SARS-CoV-2 nucleocapsid  protein IgG | Elecsys Anti-SARS-CoV-2 S ≥0.8 IU/ml | 78% | 98% | 100% | 76%  69.4 %- Anti CD38 Treatment | Overall 53 % of patients reported side-effects | Lower seroconversion rates in daratumumab-treated patients (69% vs.81%, p=0.08) | [25] |
| Lim SH,J  Lancet Haematol | Lymphoma | BNT162b2(Pfizer-BioNTech)  and AZD1222(Oxford–AstraZeneca) | 119 Patients/ 150 Controls | Negative anti-SARS-CoV-2 nucleocapsid  protein IgG | Qualified electrochemiluminescent Anti-SARS-CoV-2 S assay ( (Meso Scale Discovery, Rockville, MD, USA) >0.55 BAU/ml | 84.9% | 100% | 100% HL  81% B cell NHL | N/A | N/A | Lower seroconversion rates in patients who received systemic anti-lymphoma therapy after the second vaccine dose (p<0.0001) for BNT162b2 vaccine | [11] |
| Tzarfati KH,  Am J Hematol | NHL, HL, MM, CLL, leukemia, MDS, MPN | BNT162b2(Pfizer-BioNTech) | 315 Patients/ 108 Controls | N/A  (No History of COVID-19) | Liaison SARS-CoV-2 S1/S2 IgG test (DiaSorin, Saluggia, Italy): ≥12 AU/ml | 74.6% | 99% | 77% | 44.5%  26.6 %- Anti CD 20 Treatment | N/A | Older age (p< 0.001, higher lactate dehydrogenase (p=0.02), and number of treatment lines(p< 0.001) correlated with lower seropositivity  ALC (p< 0.001), total globulin level( p=0.002, and time from last treatment to vaccination (p< 0.001)correlated with higher seropositivity likelihood and antibody titers | [26] |
| Parry, H.  *Blood Cancer J* | CLL | BNT162b2(Pfizer-BioNTech)  and AZD1222(Oxford–AstraZeneca) | 55 Patients/ 93 Controls | Negative anti-SARS-CoV-2 nucleocapsid  protein IgG | Serum Samples--Elecsys^®^ Anti-SARS-CoV-2 S ECLIA  DBS- SARS-CoV-2 spike- specific antibody responses: ≥0.8 | 75% - Serum samples  71%- DBS | 100 % - Serum samples  97%-DBS | 77.7% | 12.5% - BTK inhibitors treatment | N/A | Lower seroconversion rates in patients treated with BTK inhibitors after the second vaccine dose (OR: 0.05; 95% CI: 0.004–0.58, *p* = 0.016)  Lower seroconversion rates in patients IgA deficiency after the second vaccine dose (IgA: OR 9.1; 2–42, *p* = 0.005) | [16] |
| Terpos E, *Blood Cancer J*. | MM | BNT162b2(Pfizer-BioNTech)  and AZD1222(Oxford–AstraZeneca) | 276 Patients (MM 213 , SMM 38, and MGUS =25)  / 226 Controls | Neutralizing  Antibodies Against SARS-CoV-2 | Neutralizing antibody assay Genscript:  ≥30% positive; ≥ 50% clinically relevant | 71% | 90.3% | 94.2% in MM patients | 54.5% -Anti CD38Treatment | 21% /31.6 % | Lower Nab reponses in patients treated with  combinations based on belantamab mafodotin (OR 7.6, 95% CI: 1.4–42.4 p = 0.021) and lymphopenia (OR: 2.1, 95% CI: 1.0–4.5, p = 0.048) after the second vaccine dose | [17] |
| Stampfer SD, *Leukemia*. | MM | BNT162b2(Pfizer-BioNTech)  and mRNA-1273 (Moderna) | 103 Patients( MM= 96 and SMM=7) / 31 Controls | Anti-SARS-CoV-2  spike IgG | Anti-SARS-CoV-2  spike IgG : 50-250 IU/ml partial response,  >250 IU/ml clinically relevant response | 67% | %100 | 100% | 67% | N/A | Lower antibody levels in MM patients receiving steroids (p = 0.0354)  Higher antibody levels in  MM patients with a complete response (CR) than those who were not in CR (p = 0.0027) | [18] |
| Ghione P, *Blood*. | Lymphoma | BNT162b2(Pfizer-BioNTech)  , mRNA-1273 (Moderna) and Ad26.COV2.S (Johnson and Johnson) | 86 Patients/ 201 Controls | Negative anti-SARS-CoV-2 nucleocapsid  antibody | The Platelia SARS-CoV-2 Total Antibody assay BioRad ≥1.0 | 41.8% | 98% | 88% | 11% (B cell directed treatment < 9 mo prior) | N/A | Lower seroconversion in patients with BCL that were actively receiving or within 3 months of completing B-cell depleting therapies 4/41 (9.7%) | [27] |
| Rahav G, *EClinicalMedicine* | MM, MDS, CLL and NHL | BNT162b2(Pfizer-BioNTech) | 418 Patients/ 272 Controls | N/A  (No History of COVID-19) | Anti–SARS-CoV-2 Spike IgG ELISA: ≥1.1 | 64.8% | 98.8% | N/A | N/A | 11.7%/38.8% | Higher seroconversion rates in patients with MM(79.7%) than in patients with CLL/NHL(51%) and MDS(60.5%) . | [28] |
| Aleman A, *Cancer Cell* | MM | BNT162b2(Pfizer-BioNTech)  and mRNA-1273 (Moderna) | 44 Patients/ 12 Controls | N/A | SeroKlir Kantaro SARS-CoV-2 IgG test: N/A | 61.3% | 100% | N/A | 63.1% - Anti CD38 Treatment | N/A | Fewer patients on active anti-BCMA bispecific therapy (2/6, 33%) or anti-CD38 antibody therapy (13/19, 68%) mounted SARS-CoV-2-specific CD4^+^ T cell responses | [29] |
| Bergman P, EBioMedicine | CLL | BNT162b2(Pfizer-BioNTech) | 79 Patients/ 78 Controls | N/A  (No History of COVID-19) | Elecsys Anti-SARS-CoV-2 S : ≥0.8 IU/ml | 63.3% | 100% | 84.6% | 88.9%- Anti CD20 Treatment  26.9% - BTK inhibitors treatment | N/A | Lowest seroconversion rate were found in patients with the ongoing BTK inhibitors treatment group(7/26) | [30] |
| Bitoun S, J Hematol Oncol | MM | BNT162b2(Pfizer-BioNTech) | 37 Patients/ 28 Controls | Negative anti-SARS-CoV-2 nucleocapsid  antibody | Elecsys Anti-SARS-CoV-2 S : ≥0.4 IU/ml | 88.9% | 96.4% | 100% | 66.6 - Anti CD38 Treatment | N/A | Progressive or stable MM was associated with a worse response (3/3 vs. 0/24; p = 0.0003) | [31] |
| Chung DJ, *Blood Cancer Discov* | Leukemia, Lymphoma and MM | BNT162b2(Pfizer-BioNTech)  and mRNA-1273 (Moderna) | 551 Patients(157 Leukemia, 173 Lymphoma and 221 MM) / 69 Controls | Anti–SARS-CoV-2 spike IgG antibody | Anti–SARS-CoV-2 spike IgG immunoassay : ≥50.0 AU/mL | 51.4% | 100% | N/A | N/A | N/A | Lower seroconversion rates in patients treated with  BTK inhibitors, venetoclax, anti-CD20–directed therapies, and anti-CD38/BCMA–directed therapies. | [20] |
| Jurgens EM, Am J Hematol | Lymphoma | BNT162b2(Pfizer-BioNTech)  and mRNA-1273 (Moderna) | 67 Patients/ 35 Controls | Negative anti-SARS-CoV-2 nucleocapsid  antibody | Anti–SARS-CoV-2 Spike IgG: OD450 ≥3 | 61.1% | 100% | N/A | 60%- BTK inhibitors treatment | N/A | Higher seroconversion rates in patients on active BTKi monotherapy (6/10) | [32] |
| Malard F, Blood Cancer J | MM, NHL, lymphoid and myeloid malignancy | BNT162b2(Pfizer-BioNTech) | 196 Patients/ 30 Controls | N/A  (No History of COVID-19) | SARS-CoV-2 IgG II Quant (Abbott, Rungis, France) : ≥3100 UA/mL | 46.7% | 87% | N/A | N/A | N/A | Male gender (OR: 0.126, 95% CI:0.022–0.709, *p* = 0.02) and ongoing chemotherapy (OR: 0.146, 95% CI:0.025–0.866, *p* = 0.03) were associated with a significantly decreased probability of achieving the defined protective anti-S IgG level after two BNT162b2 dose | [34] |
| Marchesi F, Br J Haematol | CML, MPN, MM and NHL | BNT162b2(Pfizer-BioNTech) | 182 Patients/ 36 Controls | LIAISON® SARS-CoV-2 S1/S2 IgG by DiaSorin®, Saluggia, Italy. | Liaison Anti SARS-CoV-2 S1/S2 IgG: ≥15 U/m | 61.5% | 100% | N/A | 22.8%- Anti CD20 Treatment  69.2%- Anti CD38 Treatment | N/A | Anti-S IgG titreş and neutralising antibodies were significantly correlated in the whole study population (r = 0·85, *P* < 0·001). | [35] |
| Marchesi F, Leukemia | NHL | BNT162b2(Pfizer-BioNTech) | 68 Patients/ 36 Controls | Anti SARS-CoV-2 S1/S2 IgG | Liaison Anti SARS-CoV-2 S1/S2 IgG: ≥15 U/m | 26.1% | 100% | 46.1% | 20.5%- Anti CD20 Treatment | N/A | N/A | [21] |
| Perry C, Blood Adv | NHL | BNT162b2(Pfizer-BioNTech) | 149 Patients/ 65 Controls | Negative anti-SARS-CoV-2 nucleocapsid  antibody | Elecsys Anti-SARS-CoV-2 S : ≥0.8 IU/ml | 49% | 98.5% | 89.2% | 39.6%-Anti CD20 Treatment | N/A | Shorter time since exposure to anti-CD20 Abs (OR: 1.092, 95% CI: 1.04-1.14) and lower ALC (OR: 2.82, 95% CI: 1.07-7.4)were significant predictors of a negative serological response to anti-COVID-19 vaccine | [22] |
| Tamari R, Blood Cancer J | Hematologic Malignancies | BNT162b2(Pfizer-BioNTech)  and mRNA-1273 (Moderna) | 217 Patients/ 54 Controls | Negative anti-SARS-CoV-2 nucleocapsid  antibody | AdviseDx SARS-CoV-2 IgG II assay: ≥50 AU/ml | 87% | 100% | N/A | N/A | N/A | A significantly lower level of spike Ab (P = 0.045) was noted among patients treated with daratumumab (spike Ab median, 513 AU/mL; IQR, 226.6–1,486.9, versus those not on treatment (spike Ab median, 3297 AU/ml; IQR, 655–19,499). | [33] |

**Abbreviations:** ALC: Absolute lymphocyte count; BCL: B cell lymphoma; BCMA: B cell maturation antigen; BTK: Bruton tyrosine kinase, CML: Chronic Myeloid Leukaemia; CLL: Chronic lymphocytic leukemia; COVID-19, Coronavirus disease 2019; CT: Chemotherapy; DBS: Dried blood samples; ET:Essential thrombocythemia; HL: Hodgkin Lymphoma; MDS: Myelodysplastic syndrome; MGUS: Monoclonal Gammopathy of Undetermined Significance; MF: Myelofibrosis; MM: Multiple myeloma; MPN: Myeloproliferative neoplasms; N/A: Not applicable; NHL: Non-Hodgkin Lymphoma; PV: Polycythemia vera; RBD: receptor binding domain; SARS-CoV-2: Severe Acute Respiratory Syndrome Coronavirus-2; SMM: Smoldering Multiple Myeloma; WM: Waldenstrom macroglobulinemia
